# Surface EMG in China: a report on the 2023 surface EMG symposium

**DOI:** 10.3389/fresc.2024.1353564

**Published:** 2024-02-14

**Authors:** Ping Zhou

**Affiliations:** University of Health and Rehabilitation Sciences, Qingdao, China

**Keywords:** surface EMG, symposium, workshop, rehabilitation, China

## 1 Background

**Figure 1:**
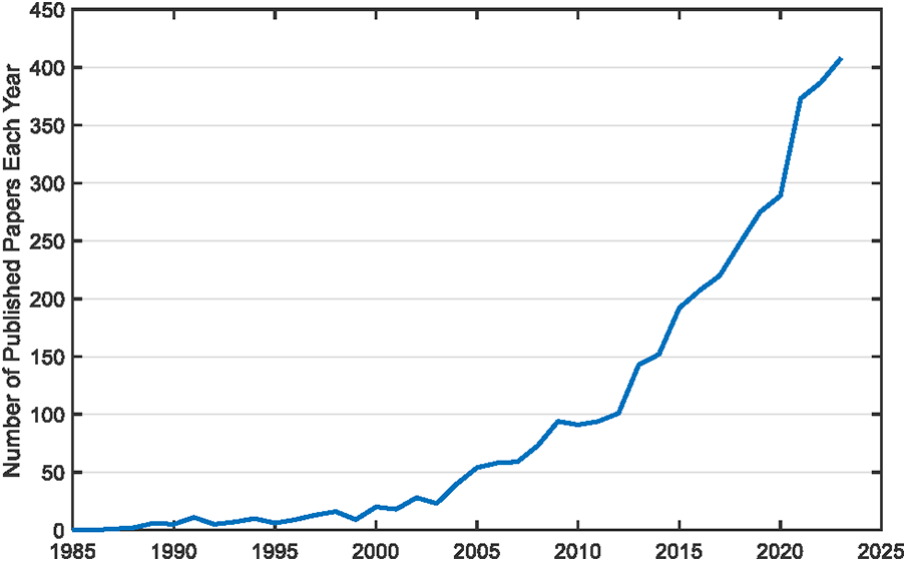
Rate of publications by authors with Chinese affiliations per year, based on a PubMed search using the following keywords contained in the title or abstract in the last 40 years: surface electromyography OR surface electromyogram OR surface EMG OR sEMG OR HDEMG OR HDsEMG.

Surface electromyography (EMG) is one of the most information-rich bioelectric signals with a wide range of applications, including in medical research, clinical diagnosis, rehabilitation, sports science, and ergonomics. There has been dramatic growth in surface EMG research over recent decades in China, due in part to continual economic development and investment in research. The rapid pace of surface EMG research is exemplified by the exponential increase in the number of publications over the past 40 years (Figure 1). The trend is also demonstrated by a considerable increase in the production of novel surface EMG equipment. Most notably, sales of high density surface EMG devices in China have increased almost 10-fold during the past 10 years (Guotao Li, personal communication, January 23, 2024; Xiaodong Liu, personal communication, January 24, 2024). As the growth of surface EMG-related research and applications in China accelerates, there is a need for more academic exchange, technical training, and knowledge dissemination.

## 2 Introduction of the symposium

This short communication introduces a recent symposium on surface EMG held in China. The symposium took place at the Hongdao International Convention and Exhibition Center in Qingdao city on November 24–25, 2023, and was chaired by the author of this communication. The theme of the symposium was “applications of surface EMG in clinical and rehabilitation practices”. More than 250 people from across academic, medical, and industrial sectors gathered together for in-depth discussions on the latest research, cutting-edge technologies, and novel applications of surface EMG. The symposium was hosted by the University of Health and Rehabilitation Sciences (Qingdao), and co-organized by the Shandong Provincial Biomedical Engineering Society and the Rehabilitation Engineering Branch of the Chinese Biomedical Engineering Society. The symposium was sponsored by Advictor Beijing Information Technology Co., Ltd. and the University of Health and Rehabilitation Sciences.

## 3 Summary of presentations

Twenty-six researchers presented research related to surface-EMG (Table 1). The talks touched on a number of applications, and reflects the current state of the art of surface EMG research in China. The majority (>70%) of presenters were university professors with a background in biomedical engineering. Three presentations were given by physician researchers. The presentations can be categorized into the following research areas.

• The first research area related to decoding of surface EMG and development of advanced myoelectric control for prostheses or rehabilitation robotics. These presentations highlighted the use of various approaches such as myoelectric pattern recognition, machine learning, deep learning, and surface EMG decomposition and projection. In particular, presented works focused on improving the reliability, adaptability, and usability of myoelectric control.
• The second research area related to innovation of surface EMG signal processing for extraction of meaningful physiological information. This included advances in high density surface EMG decomposition, motor unit number estimation, muscle innervation zone detection, muscle fiber conduction velocity estimation, and nonlinear dynamic analysis of surface EMG. In addition, presentations also expanded upon applications of these methods for examination of muscle performance, such as detection of muscle activity onset, prediction of muscle force, and assessment of muscle fatigue.
• The third research area related to neuromuscular assessment of neurological injuries or neuromuscular disorders using conventional, linear, or 2-dimensional high density surface EMG. High density surface EMG was a particular focus as it provides both temporal and spatial information of whole muscle and motor unit activity. The studied neuromuscular disorders included stroke, spinal cord injury, and cerebral palsy, etc. The presenters also discussed pathological mechanisms at the whole muscle or motor unit level that may contribute to disabilities.
• Three physician researchers discussed applications of surface EMG in their clinical practice. They presented case studies, demonstrating how surface EMG can provide important qualitative information to aid in diagnosis, prognosis, and evaluation of treatments in various neuromuscular disorders, such as radiculopathy, peripheral neuropathy, and degenerative osteoarthropathy.

**Table 1:** A list of oral presentations (in chronological order) at the 2023 surface EMG symposium, Qingdao, China.

Presenter	Affiliation	Title of presentation
Guanglin Li, PhD	Shenzhen Institutes of Advanced Technology, Chinese Academy of Sciences, Shenzhen	Application of high density surface EMG in neuromuscular function assessment
Jian Wang, PhD	Zhejiang University, Hangzhou	Surface EMG assessment of muscle fatigue: detection technology and application prospects
Wensheng Hou, PhD	Chongqing University, Chongqing	Surface EMG assessment of motor function in infants and young children
Ning Jiang, PhD	West China Hospital, Sichuan University, Chengdu	A 10-year journey of intelligent limb prosthesis research: robot-human integration for functional regeneration of amputees
Yong Hu, PhD	Shenzhen Hospital, University of Hong Kong, Shenzhen	Surface EMG spatial matrix for long-term wearing of intelligent prosthetic hands
Jin Xu, PhD	Xi'an Jiao Tong University, Xi'an	Surface EMG based real-time recognition of multiple gestures and system development
Rong Song, PhD	Sun Yat-sen University, Guangzhou	Myoelectric control methods for rehabilitation robots
Le Li, PhD	Northwestern Polytechnical University, Xi'an	Neuromuscular function assessment after spinal cord injury based on linear array EMG
Pengxu Wei, MD	National Research Center for Rehabilitation Technical Aids, Beijing	EMG-based walking movement pattern analysis—physiological interpretation and application value of matrix decomposition algorithms
Xu Zhang, PhD	University of Science and Technology of China, Hefei	Intelligent neuromuscular interface and applications
Bo Yao, PhD	Institute of Biomedical Engineering, Chinese Academy of Medical Sciences, Tianjin	Surface EMG examination of scoliosis
Ya Zong, MD	Ruijin Hospital, Shanghai Jiao Tong University, Shanghai	Surface EMG: new technologies and applications in rehabilitation department
Chuanxin Niu, PhD	Ruijin Hospital, Shanghai Jiao Tong University, Shanghai	Quantitative analysis of muscle group coordination during upper limb extensive movement after stroke
Lin Xu, PhD	Shanghai University of Science and Technology, Shanghai	Analysis of surface EMG signals during vibration training with alternating loads
Ping Zhou, PhD	University of Health and Rehabilitation Sciences, Qingdao	Innovations in surface EMG technology and its application in neuromuscular rehabilitation
Zhiyuan Lu, PhD	University of Health and Rehabilitation Sciences, Qingdao	Assessment of upper limb muscle function and motor patterns after stroke
Chengjun Huang, PhD	University of Health and Rehabilitation Sciences, Qingdao	Exploration of muscle innervation zones using surface electrode array EMG
Tianzhe Bao, PhD	University of Health and Rehabilitation Sciences, Qingdao	Towards higher interpretation, adaptation, and reliability in upper-limb myoelectric control
Raymond Kai-Yu Tong, PhD	The Chinese University of Hong Kong, Hong Kong	Exploring the efficacy of EMG-driven robotic hand systems in the rehabilitation of central and peripheral nerve injuries
Ke Li, PhD	Shandong University, Ji'nan	Dynamic EMG network based on nonlinear dynamics
Xiang Chen, PhD	University of Science and Technology of China, Hefei	Force prediction based on high-density surface EMG and exploration of its clinical application
Chenyun Dai, PhD	Shanghai Jiao Tong University, Shanghai	Exploring the interpretability of task-related and individual-related components in EMG
Xiaodong Zhang, PhD	Xi'an Jiao Tong University, Xi'an	Myoelectric fine sensing method and its application to rehabilitation robot
Dapeng Yang, PhD	Harbin Institute of Technology, Harbin	Intelligent bionic dexterous hand and its manipulation technology
Weijun Gong, MD	Beijing Rehabilitation Hospital, Beijing	Application of surface EMG for diagnosis and treatment of degenerative osteoarthropathy
Yang Zheng, PhD	Xi'an Jiao Tong University, Xi'an	Decoding finger movements based on EMG decomposition

## 4 High density surface EMG decomposition workshop

In addition to scientific presentations, a workshop on high density surface EMG decomposition was held on the afternoon of November 25th. Dr. Maoqi Chen (the University of Health and Rehabilitation Sciences, Qingdao) delivered a training course on the progressive FastICA peel-off (PFP) method for high density surface EMG decomposition (1, 2). Dr. Chen shared an implementation program of the PFP algorithms (written in MATLAB) with all workshop participants. The training content included explanations of the rationale and principles of the PFP algorithms, instructions for using the PFP decomposition program, hands-on practice for decomposing high density surface EMG data, and a final period of Q&A and follow-up interactions. In particular, participants were trained on how to judge and accept reliable motor units, while avoiding spurious ones or other pitfalls (3). This is extremely important for practical applications, especially for novice or lay users. The workshop provided useful theoretical, technological, and software support for the participants interested in high density surface EMG research. A total of 128 trainees from different backgrounds (academic scientists, clinicians, engineers, graduate students, R&D staff) attended the workshop. It was emphasized that the PFP decomposition program delivered in the workshop should be used for research (non-commercial) purposes only.

## 5 Conclusion and future prospectives

The 2023 Surface EMG Symposium provided an interdisciplinary academic exchange platform for Chinese researchers in the field of surface EMG in academic, clinical, and rehabilitation practice. The symposium provided a forum for considerable discussion and sharing of ideas. The meeting generated much interest in developing new scientific collaborations among the participants. The symposium received more presentation requests than could be accommodated in a two day period, highlighting the strong interest in surface EMG in the research community. Given this strong interest, it is expected that similar symposium may occur regularly, perhaps every two years. Although this symposium covered a broad range of topics, there are other important topics or issues that were not addressed. These include recent advances in the manufacture of surface EMG electrodes and EMG data acquisition devices (4–6) and novel applications of surface EMG, including for pelvic floor examination and treatment (7). In addition, although surface EMG has achieved significant development in recent decades, barriers limiting widespread use in clinical and rehabilitation practice remain (8, 9). We hope these and other matters pertaining to surface EMG research can be addressed in future symposia.

## Data Availability

The original contributions presented in the study are included in the article/Supplementary Material, further inquiries can be directed to the corresponding author.
